# NLRP3‐Caspase‐1 Axis in Human Adipose Tissue Crown‐Like Structures: A Potential Mediator of Inflammation and the Effects of Bariatric Surgery

**DOI:** 10.1002/iid3.70312

**Published:** 2026-02-05

**Authors:** Ville A. Palomäki, Juha P. Väyrynen, Vesa Koivukangas, Sanna Meriläinen, Tuomo J. Karttunen, Petri Lehenkari

**Affiliations:** ^1^ Department of Surgery Kuopio University Hospital Kuopio Finland; ^2^ Cancer and Translational Medicine Research Unit, Medical Research Center Oulu Oulu University Hospital and University of Oulu Oulu Finland; ^3^ Department of Surgery Oulu University Hospital Oulu Finland

**Keywords:** caspase‐1, crown‐like structures, inflammation, macrophage, NLRP3, obesity

## Abstract

**Background:**

Obesity‐related comorbidities, such as type 2 diabetes, are associated with chronic inflammation mediated by macrophages. This inflammation is characterized by the accumulation of macrophages in crown‐like structures (CLS) surrounding dead adipocytes within adipose tissue. In a recent study, we reported a significant reduction in CLS‐associated macrophages following bariatric surgery. The NLRP3 inflammasome and its downstream effector, Caspase‐1, are well‐established mediators of metabolic dysfunction and inflammation in obesity.

**Methods:**

Immunohistochemical single‐ and multiplex staining of subcutaneous adipose tissue (SAT) samples from patients with obesity was performed to characterize the detailed distribution of NLRP3 and Caspase‐1. The samples were collected during gastric bypass surgery and at a 1‐year follow‐up.

**Results:**

Both NLRP3 and Caspase‐1 were expressed by CD68‐positive macrophages in SAT including both single macrophages and those forming CLS. A reduction in the total number of SAT macrophages expressing these proteins was detected following gastric bypass. Furthermore, NLRP3 and Caspse‐1 were observed in the endothelium of vascular structures.

**Conclusions:**

Our findings demonstrate that both CLS‐forming and single cell macrophage populations in human adipose tissue express NLRP3 and Caspase‐1. The study highlights the significance of the NLRP3 inflammasome macrophages in the pathogenesis of adipose tissue inflammation in obesity. The previously reported abundance of CLS in untreated obesity and their reduction after bariatric surgery suggest that the NLRP3‐Caspase‐1 axis within CLS may serve as an important mediator of the beneficial metabolic effects associated with bariatric surgery.

## Graphical abstract

NLRP3 and Caspase‐1 are mainly expressed in subcutaneous adipose tissue macrophages and are highly enriched in CLS. The NLRP3‐caspase‐1 axis in CLS may be a key mediator of obesity‐associated inflammation of adipose tissue and may explain some of the beneficial metabolic and anti‐inflammatory effects of bariatric surgery.

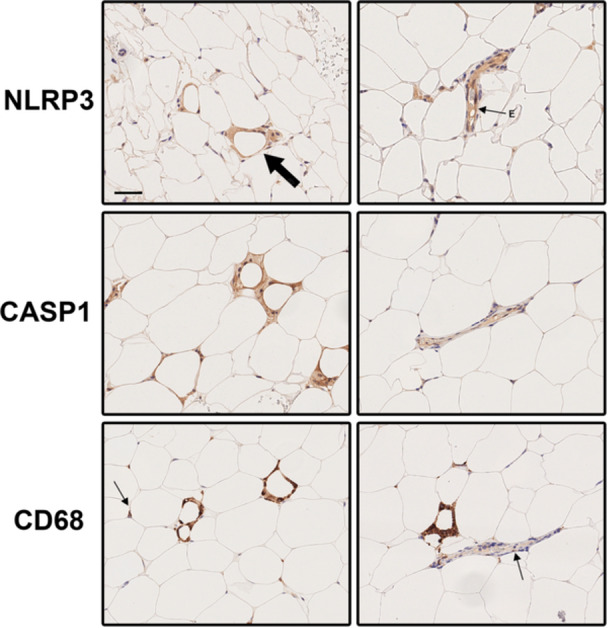

**Abbreviations**
CLS: crown‐like structures
IL‐18: Interleukin‐18
IL‐1β: Interleukin‐1β
NLRP3: nucleotide‐binding oligomerization domain‐like receptor‐3
SAT: subcutaneous adipose tissue

## 1 Introduction

Obesity and its associated comorbidities are a major health concern worldwide [1, 2]. Low‐grade inflammation within excess adipose tissue is increasingly recognized as a key contributor to obesity‐related comorbidities [3, 4]. During weight gain, adipocytes enlarge and the adipose tissue is infiltrated by macrophages promoting both local and systemic chronic inflammation [5, 6]. The macrophage infiltration into adipose tissue, as well as cytokines such as interleukin‐1β (IL‐1β) and interleukin‐18 (IL‐18) released in the process, are critical factors in the development of insulin resistance and type 2 diabetes [7]; G. S [8, 9, 10, 11, 12].

Cinti et. al. [13] demonstrated that adipose tissue inflammation is characterized by the death of hypertrophic adipocytes and macrophage sequestering of the leftover debris and lipid droplets, forming a distinct histological feature known as CLS. In human obesity, approximately 15%–20% of SAT infiltrating macrophages are found in these aggregates. The presence of CLS is associated with inflammation and insulin resistance [14, 15]. Interestingly, bariatric surgery, which is well‐known for its beneficial effect on metabolic dysfunction, selectively reduce the number of CLS‐associated macrophages in adipose tissue of subjects with obesity [14, 15, 16].

In obese mice, CLS‐associated macrophages contain a nucleotide‐binding oligomerization domain‐like receptor 3 (NLRP3) inflammasome complex, which, with subsequent activation of Caspase‐1 enzyme, mediate the production of IL‐1β and IL‐18 proinflammatory cytokines [12, 17]. The NLRP3 inflammasome serves as a cellular sensor for local danger signals such as extracellular ATP from injured cells, adjacent necrosis, bacterial molecular patterns and free fatty acids [18, 19, 20, 21]. Obesity, inflammation and impaired glucose metabolism are associated with increased expression of NLRP3 and related genes in human and mice SAT, as well as with Caspase‐1‐mediated release of IL‐1β and IL‐18 [12, 22, 23, 24].

In murine models, NLRP3 and Caspase‐1 are enriched in immune cells and colocalize within adipose tissue CLS [12]. However, the precise distribution of these proteins in the adipose tissue of human subjects with obesity remains unclear. Pahwa et al. [25] recently reported that Caspase‐1 expression, as assessed by immunohistochemistry, was more abundant in SAT of subjects with nascent metabolic syndrome compared to healthy controls. Furthermore, Caspase‐1 expression was correlated with various markers of metabolic risk and inflammation as well as certain inflammasome triggering danger‐ or pathogen‐associated molecular patterns. Nevertheless, the study did not provide specific details regarding the histological localization of the immunostaining.

We recently quantified CLS‐associated macrophage and the diffuse single‐cell macrophage populations in the SAT of subjects with obesity. Our findings revealed that only the number of CLS macrophages decreased following bariatric surgery, suggesting distinct functional roles for these two populations [15]. We hypothesized that NLRP3 and Caspase‐1 might mediate the inflammatory response elicited by adipose tissue macrophages in obesity. Our results demonstrate that these proteins are predominantly expressed in adipose tissue macrophages, with minor expression in the endothelium of vascular structures. Notably, a constant expression of NLRP3 and Caspase‐1 was observed in the CLS, emphasizing the importance of this “bariatric surgery responsive pool” of macrophages in the development of an inflammatory state of adipose tissue in obesity.

## 2 Material and Methods

### 2.1 Subjects

Sixty subjects with obesity scheduled for laparoscopic gastric bypass surgery in Oulu, Finland, in 2014–2019 volunteered in the study focusing on the effect of bariatric surgery on inflammation and osteoarthritis symptoms. For anthropometrics and inclusion criteria, see Palomäki et al. [15]. In short, the subjects had BMI over 40 kg/m^2^ or BMI over 35 kg/m^2^ and obesity‐associated comorbidity or BMI over 30 kg/m^2^ and type 2 diabetes which was not adequately managed with conservative treatment. Prior to surgery, all subjects underwent a three‐ to 5‐week very‐low‐calorie diet.

### 2.2 Tissue Samples and Immunohistochemistry

Approximately 1 cm^3^ subcutaneous adipose tissue biopsy from the periumbilical area was obtained during the gastric bypass surgery or under local anesthesia at 1 year after the surgery, fixed in formalin, and embedded in paraffin. After data loss (refusals, dropouts etc.), 39 paired samples were acquired. The specimens were sectioned at 5 µm and stained with hematoxylin & eosin (H&E) or reserved to immunohistochemistry.

Immunohistochemistry was performed with Leica BOND RX Automated Research Stainer (Leica Biosystems, Buffalo Grove, IL, USA). Single‐plex immunohistochemistry for CD68, NLRP3 and Caspase‐1 was performed for all 39 paired samples. For staining, BOND Polymer Refine Detection System (DS9800; Leica Biosystems, Buffalo Grove, IL, USA) was used with 3,3′‐Diaminobenzidine as a chromogen. For macrophage staining, mouse monoclonal Anti‐Human CD68 (PGM1; M087601‐2, Dako Agilent, Santa Clara, CA, USA; dilution 1:200) was used for 30 min at room temperature (RT) after 20 min pretreatment in Bond Epitope Retrieval Treatment 2 (100 C; Leica Biosystems). Rabbit polyclonal Anti‐Human NLRP3 (Cat no:19771‐1AP, Proteintech, Rosemont, IL, USA; dilution 1:2000) for 30 min at RT after pretreatment in Bond Epitope Retrieval Treatment 1 (Leica Biosystems). For CASP1 we used polyclonal rabbit Caspase‐1 antibody (ab62698, lot GR104507‐27; Abcam, Boston, MA, USA; dilution 1:300) for 2 h at RT after pretreatment in Bond Antigen Retrieval Solution 1 for 30 min used. CLS density was assessed as described elsewhere [15].

Multiplex immunohistochemistry was performed in a subset of 18 specimens, selected to represent variation in the amount of macrophage populations and included cases with high or low baseline CLS density (14 specimens), and the 1‐year follow‐up samples of cases with initially high baseline CLS density (4 specimens) who underwent laparoscopic gastric bypass. For multiplex immunohistochemistry, a previously described, cyclic method was utilized [26], where one marker was stained per cycle, and the slide was subsequently scanned and prepared for the next staining cycle. Each staining cycle followed the protocol optimized for single‐plex immunohistochemistry with the following exceptions: 1) ImmPACT® AMEC Red (Vector Laboratories, Newark, CA, USA; incubation time 20 min) was used as the chromogen instead of DAB; and 2) the sections were not dehydrated after the staining and cover slips were mounted with an aqueous mounting medium. Between the cycles, the coverslips were removed by soaking the slides in distilled water. AMEC was removed by washing the slides in ethanol, followed by antibody stripping in heated (100°C) citrate buffer (Leica BOND Epitope Retrieval Solution 1, Leica Biosystems). The order of antibodies in multiplex staining was 1) CD68, 2) CASP1, 3) NLRP3. The correspondence of the staining patterns between single‐plex and multiplex immunohistochemistry was visually confirmed. The completeness of the removal of previous antibody between the staining cycles was evaluated by replacing the subsequent antibody with antibody diluent only.

### 2.3 Image Reconstruction and Analysis

Stained SAT sections were scanned with Leica Aperio AT2 (Leica Biosystems, Buffalo Grove IL USA) at 40x magnification. A structure was deemed CLS when immunostained macrophage aggregate circularly surrounded at least half of a presumably dead adipocyte or a lipid droplet [27]. We defined a CLS remnant as a glomerate of multiple macrophages found among the adipocytes without any recognizable lipid droplet left. Immunohistochemical staining was quantified using QuPath 0.5.1, employing a pixel classifier trained on a minimum of 15 representative images. Subsequently, the classifier was utilized to determine the area of immunopositive and immunonegative tissue within annotated adipocyte regions of whole‐slide sections, with or without the exclusion of vascular structures. The method for adipocyte density analysis is described elsewhere [15].

### 2.4 Statistical Methods

Statistical analyses were conducted with SPSS for Windows (version 25, IBM Corp., Armonk, NY, USA). Normality of data was estimated with Shapiro‐Wilk test. Difference in paired values was expressed as mean and the 95% confidence interval (CI). Wilcoxon Signed Rank were used for estimation of repeated measures. For bivariate correlation analysis, Pearson correlation coefficient (r) or Spearman's correlation coefficient (ρ) were calculated for parametric and non‐parametric variables, respectively. Two‐tailed p‐values are presented.

## 3 Results

### 3.1 Immunostaining for NLRP3 and Caspase‐1 Is Predominantly Found in Macrophages in SAT

**Figure 1:**
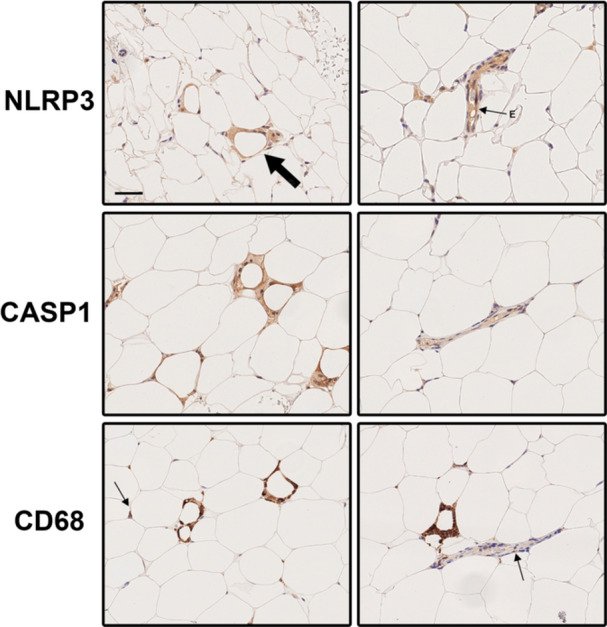
Immunohistochemical staining of NLRP3, Caspase‐1, and CD68 was performed on subcutaneous adipose tissue samples from subjects with obesity. NLRP3 and Caspase‐1 were enriched in crown‐like structures (CLS) and colocalized with macrophage CD68 staining, except in the endothelium. Left column: similar staining was observed in single cell macrophages (thin arrow) and CLS (thick arrow). Right column: NLRP3 and Caspase‐1 were also found in the endothelium (thin arrow + E) of adipose tissue vascular structures. However, CD68 staining in vascular structures was more scattered, suggesting that these cells were either circulating or had recently emigrated from the vessel (thin arrow). CASP1; Caspase‐1, NLRP3; nucleotide‐binding oligomerization domain‐like receptor‐3. Scale bar 50 µm.

Single‐plex stainings for NLRP3 and Caspase‐1 were thoroughly reviewed on whole‐slide sections. Expression of both markers was predominantly observed in single mononuclear cells dispersed among adipocytes and in cells forming the CLS. The staining pattern closely resembled that of the macrophage marker CD68 (Figure 1, left panel). Notably, NLRP3 and Caspase‐1 staining was particularly intense within the CLS (Figure 1). However, NLRP3 and Caspase‐1 antibodies also stained the endothelium of the perfusing blood vessels in the adipose tissue (Figure 1, right panel, small arrow + E). The endothelium was negative for CD68, and only infrequent positively stained cells were identified within the vessels (Figure 1, right panel, small arrow).

**Figure 2:**
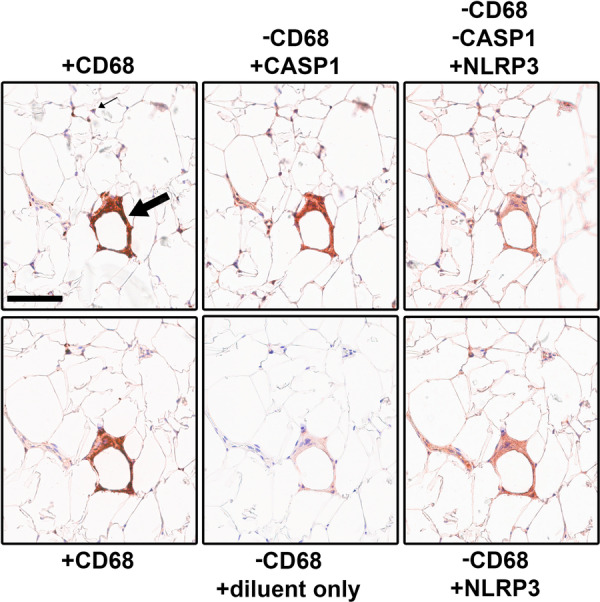
Multiplex staining of subcutaneous adipose tissue sections from subjects with obesity for CD68, Caspase‐1, and NLRP3 confirmed the localization of the NLRP3‐Caspase‐1 axis in macrophages with minor localization in the endothelium. Representative image. Upper row: The same section was consecutively stained for (+) CD68, Caspase‐1 and NLRP3 with removal (−) of the antibodies between the steps. Immunostaining overlapped in crown‐like structures (CLS) (large arrow) and is found in the same single‐cell macrophages (small arrow). Lower row: An adjacent section was similarly treated with omission of second step antibody to assess the efficiency of antibody removal procedure. CASP1; Caspase‐1, NLRP3; nucleotide‐binding oligomerization domain ‐like receptor‐3. Scale bar 100 µm.

Next, multiplex staining was conducted to investigate the colocalization of NLRP3 and Caspase‐1 with the macrophage marker CD68. Negative controls, omitting the secondary antibody, revealed only minor residual staining in areas of high signal intensity (Figure 2), confirming the reliability of the multiplex technique. As shown in Figure 2, all positive NLRP3 or Caspase‐1 staining was observed in CD68 expressing single macrophages and CLS macrophages, excluding endothelial cells. No discernible staining for NLRP3 or Caspase‐1 was detected in adipocytes.

**Figure 3:**
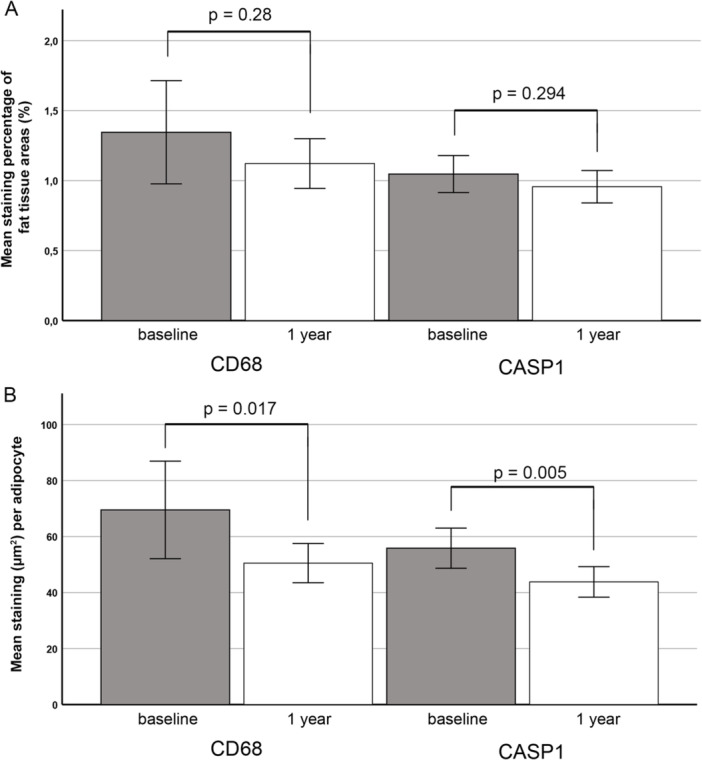
Quantitative analysis of CD68 and Caspase‐1 expression in paired subcutaneous adipose tissue samples at baseline and one year after gastric bypass surgery. Immunostained area within adipocyte regions was quantified using the pixel classifier feature of QuPath 0.5.1. (A) The percentage of total adipose tissue area positive for CD68 staining was quantified. For Caspase‐1 analysis, irregularly shaped vascular structures with endothelial staining were excluded. A declining trend in the amount of staining was observed for both CD68 (paired samples *N* = 33) and Caspase‐1 (*N* = 37); however, these decreases were not statistically significant (Wilcoxon Signed Ranks Test (*p* = 0.28 and *p* = 0.294, respectively). (B) CD68 staining and Caspase‐1 staining (excluding vascular structures) within adipose tissue areas are expressed as stained area (µm^2^) per adipocyte. Weight loss induced by gastric bypass is associated with a reduction in adipocyte diameter, leading to increased cell density. Following correction for this change in cell density, a significant reduction in both CD68 and Caspase‐1 staining per adipocyte was observed in paired samples (Wilcoxon Signed Ranks Test, *p* = 0.017 and *p* = 0.005 for, respectively). CASP1; Caspase‐1.

Paired samples, collected at baseline and 1 year following gastric bypass surgery, were analyzed for the expression of CD68 (33) and Caspase‐1 (37). The percentage of immunostained areas within delineated adipocyte regions was quantified using the pixel classifier feature of QuPath 0.5.1. For Caspase‐1 quantification, vascular structures were manually excluded from the analysis. Mean CD68 and Caspase‐1 staining decreased by 0.22 (16.6%) and 0.07 (7.0%) percentage points, respectively (Figure 3A); nevertheless, this reduction was not statistically significant. However, a reduction in mean adipocyte diameter is known to occur after gastric bypass induced weight‐loss [15]. When density correction was applied and staining areas per adipocyte were calculated, the mean stained area was found to have significantly decreased by 19.0 µm²/adipocyte (95% CI: 2.7–35) (27.3%) for CD68 and 11.7 µm²/adipocyte (95% CI: 4.2–19.2) (20.8%) for Caspase‐1 (Figure 3B). CD68 stained areas had a moderate correlation with Caspase‐1 staining both at baseline and 1‐year after the operation (Spearman's ρ = 0.552, *p* < 0.000 and Spearman's ρ = 0.507, *p* = 0.003, respectively). A microphotograph of Caspase‐1 staining in adipose tissue at baseline and 1 year after bariatric surgery is included as a Supplementary Figure.

### 3.2 Dynamics of CLS Lifespan as Seen With CD68, NLRP3 and Caspase‐1 Stainings

**Figure 4:**
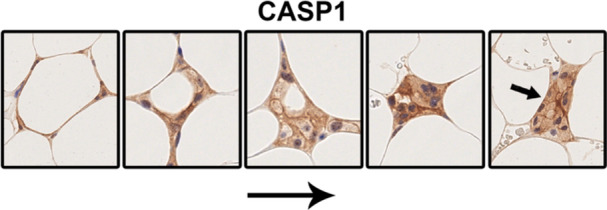
Proposed histological phases of crown‐like structures' (CLS) lifespan. From left to right: Initially, macrophages (here immunostained for Caspase‐1) sequester a presumably dead adipocyte which still retains its form. Macrophages then consume the lipid droplet and debris, leaving only a cluster of macrophages likely representing a “CLS remnant” (far right). CASP1; Caspase‐1. Images are not in scale.

CLS formation has been proposed as a mechanism for clearing debris and lipid droplets from dying adipocytes. Upon histological examination of stained sections, it became evident that CLS were present in various stages of their lifecycle within any sample exhibiting sufficient CLS formation. Figure 3 shows the presumed histological phases of CLS' lifecycle. In the early stages of CLS formation, the adipocyte often retained its original shape (Figure 4, leftmost image). In sections containing CLS, distinct macrophage clusters devoid of lipid droplets were occasionally observed (Figure 4, small arrow). These clusters could represent CLS remnants following the dissolution of lipid droplets or the peripheral regions of spherical CLS, where lipid droplets might only be visible in deeper tissue planes. Although no formal quantification of staining intensity was conducted to compare the different stages of CLS formation, it was our observation that there were no significant sequential changes (Figure 4).

## 4 Discussion

We show here that in histological specimens of SAT from subjects with obesity, NLRP3 and Caspase‐1 are predominantly expressed in single‐cell and CLS‐macrophages marked with CD68 staining. In addition, NLRP3 and Caspase‐1 staining were present in endothelial cells. These proteins and their downstream products IL‐1β and IL‐18 are pivotally involved in obesity‐related low‐grade inflammation and associated metabolic impairments, such as insulin resistance [12, 23, 28, 29, 30]. Following gastric bypass surgery, a significant decrease in positively stained areas per adipocyte were observed for both the macrophage marker CD68 and that of Caspase‐1, when endothelial staining for Caspase‐1 was excluded. Since adipocyte size is known to decrease after bariatric surgery along with the decrease of the amount of adipose tissue, this finding suggests that associated with weight‐loss, proinflammatory load from the SAT macrophages had decreased.

We previously demonstrated that gastric bypass surgery for obesity results in a specific weight‐loss independent reduction of CLS‐associated macrophages in SAT without affecting single‐cell macrophages [15]. This suggests that the decreased expression of NLRP3 and Caspase‐1 in SAT following gastric bypass surgery may primarily be due to the decrease or disappearance of CLS. The beneficial effects of gastric bypass for obesity on metabolic impairments and insulin resistance are well‐documented, and it is possible that some of these effects are mediated by alterations to NLRP3 inflammasome mediated low‐grade inflammation [31]; W [32, 33, 34]. Our findings suggest that macrophages within CLS may play a pivotal role in both the beneficial effect of bariatric surgery and the development of obesity‐related metabolic disturbances such as insulin resistance. However, due to lack of quantitation and analysis of cell type specific expression levels of NLRP3 and CASP1 we cannot exclude possibility that in addition to decrease of the CLS numbers the change of cell specific expression levels of NLRP3 and CASP1 in macrophages also contributes to beneficial effects of bariatric surgery. In our attempts to assess cell type–specific staining of NLRP3 and CASP1, visual estimation was unreliable due to heterogeneous staining, and Qupath analysis was unfeasible because cell population classification was not possible, and manual annotation was too time‐consuming.

Mocanu et al. [33] reported that bariatric surgery in rats suppressed NLRP3 inflammasome activation in visceral adipose tissue (VAT) and improved glycemic control. However, the effects on subcutaneous SAT were less pronounced. Notably, CLS density and macrophage infiltration are generally higher in VAT than SAT [14, 35]. In other words, the rat model may have limited sensitivity in detecting changes in SAT compared to human tissues. Additionally, visceral obesity is widely recognized as more detrimental to metabolic health than subcutaneous fat [35, 36]. Therefore, it is plausible that the NLRP3 inflammasome expression and CLS density in SAT are considerably lower than in VAT, particularly in individuals with metabolically unhealthy obesity [37].

In a recent study, Pahwa et al. [25] reported that Caspase‐1 staining was predominantly found in the stromal‐vascular compartment of SAT. However, they did not observe a correlation between Caspase‐1 and the macrophage marker CD68. Our current findings confirm the presence of Caspase‐1 staining in vascular structures but extend these observations by demonstrating its localization primarily within CD68‐positive macrophages. Pahwa et al. presumably used ImageJ and quantified expression levels using relative absorbance units [25, 38]. It is possible that the particular ratio of macrophages to vascular structures in their samples may have confounded the results if not separately quantified.

CLS formation in adipose tissue is reminiscent of NLRP3 and Caspase‐1‐associated programmed cell death, pyroptosis [17, 39]. The sequestration and clearance of lipid droplets is a dynamic process that can extend over several days or even a week [40, 41]. Identifying the triggers of CLS formation is of particular interest, as they may offer potential targets for therapeutic intervention to mitigate the obesity‐associated inflammation and morbidities. Likely candidates for CLS triggers include local danger signals that activate the NLRP3 inflammasome, such as extracellular ATP, free fatty acids, and pathogen‐associated molecular patterns. However, less well‐known factors, including environmental chemicals stored in adipose tissue, may also be involved.

Our study has certain limitations. Initial subcutaneous adipose tissue samples were collected during bariatric surgery. The customary preoperative low‐calorie diet, typically administered for 2–3 weeks, induces some weight loss and improves metabolic and inflammatory parameters [42]. This intervention may have altered adipose tissue macrophage populations and their inflammasome activity. Consequently, the observed macrophage inflammasome activation in obesity may not fully reflect a stable, long‐term abnormality but could be partially influenced by diet‐induced changes. In our series, data on weight loss following the preoperative diet was unavailable. However, such weight loss generally represents only a small fraction (~17%) of total postoperative weight loss [42], suggesting that its impact on adipose tissue inflammation is likely minor.

A second limitation is the absence of a normal‐weight control group. Recent studies indicate that, even after successful bariatric surgery and normalization of systemic inflammatory markers, circulating monocytes may remain partially activated potentially contributing to residual cardiovascular risk [43, 44]. To better understand the role of adipose tissue macrophages in this persistent risk, it would be valuable to compare inflammasome protein expression between healthy individuals and patients with obesity after bariatric surgery. Similarly, examining endothelial inflammasome marker expression in normal‐weight individuals would help determine whether endothelial activation represents a pathological response specifically linked to obesity, even after surgical treatment.

Future studies should establish whether inflammasome activation within the CLS and other macrophages of SAT indeed exerts systemic proinflammatory effects. Measuring local and systemic levels of IL‐1β and IL‐18, along with their correlation with CLS status and density, could provide valuable insights. Furthermore, in functional studies of adipose tissue macrophages in obesity, it is important to separate CLS‐associated macrophages from other single‐cell macrophages. The potential role of endothelial inflammasome expression should also be considered, especially if it exhibits dynamic changes in response to obesity treatment. Finally, given the reported differences between VAT and SAT, assessing CLS and inflammasome status within VAT from human subjects could offer additional valuable information. However, the acquisition of paired VAT samples in human subjects requires additional surgery, which renders it ethically challenging and was not considered in this study.

## 5 Conclusions

In conclusion, we have demonstrated that both single macrophages and those within the CLS express the NLRP3 inflammasome and Caspase‐1. Furthermore, we have shown that along with decrease of CD68 staining per adipocyte, non‐vascular staining for Caspase‐1 is decreased after bariatric surgery, and although not quantitated, NLRP3 expression shows a similar change. Since macrophages in the CLS form the dynamic component responding to bariatric surgery [15], these findings suggest that CLS‐associated macrophages, with the NLRP3 inflammasome and likely producing IL‐1β and IL‐18, contribute significantly to the chronic low‐grade inflammation associated with obesity and its related comorbidities, such as insulin resistance and type 2 diabetes. The remission of these conditions following gastric bypass may be partially attributed to a reduction in CLS‐associated NLRP3‐Caspase‐1 axis activity after the surgery.

## Author Contributions

**Ville A. Palomäki:** conceptualization, data curation, formal analysis, visualization, writing – original draft, writing – review and editing. **Juha P. Väyrynen:** investigation, methodology, formal analysis. **Vesa Koivukangas:** investigation, supervision, funding acquisition. **Sanna Meriläinen:** investigation. **Tuomo J. Karttunen:** methodology, writing – review and editing. **Petri Lehenkari:** investigation, supervision, funding acquisition, project administration, writing – review and editing.

## Ethics Statement

Oulu University Hospital Ethics Committee authorized the study (EETTMK67/2013) and informed written consent for participation in the study was acquired from the subjects.

## Conflicts of Interest

The authors declare no conflicts of interest.

## Supporting information


**Supplementary Figure:** Immunohistochemical staining of subcutaneous adipose tissue samples from the same subject was performed at baseline (A) and one year after (B) bariatric surgery using Caspase‐1 antibody. A decline in crown‐like structure numbers was observed; however, Caspase‐1 was still present in individual macrophages and in the endothelium of vascular structures. Scale bar 100 μm.

## Data Availability

Data sets generated during this study are available upon reasonable request from the corresponding author.
