# Supplementary material for: NLRP3‐Caspase‐1 Axis in Human Adipose Tissue Crown‐Like Structures: A Potential Mediator of Inflammation and the Effects of Bariatric Surgery
Source: Immun Inflamm Dis. 2026 Feb 5;14(2):e70312. doi: 10.1002/iid3.70312 (PMC12877315; doi:10.1002/iid3.70312)
Supplement: Supplementary file 1 — Supplementary Figure: Immunohistochemical staining of subcutaneous adipose tissue samples from the same subject was performed at baseline (A) and one year after (B) bariatric surgery using Caspase‐1 antibody. A decline in crown‐like structure numbers was observed; however, Caspase‐1 was still present in individual macrophages and in the endothelium of vascular structures. Scale bar 100 μm. [file IID3-14-e70312-s001.pdf]

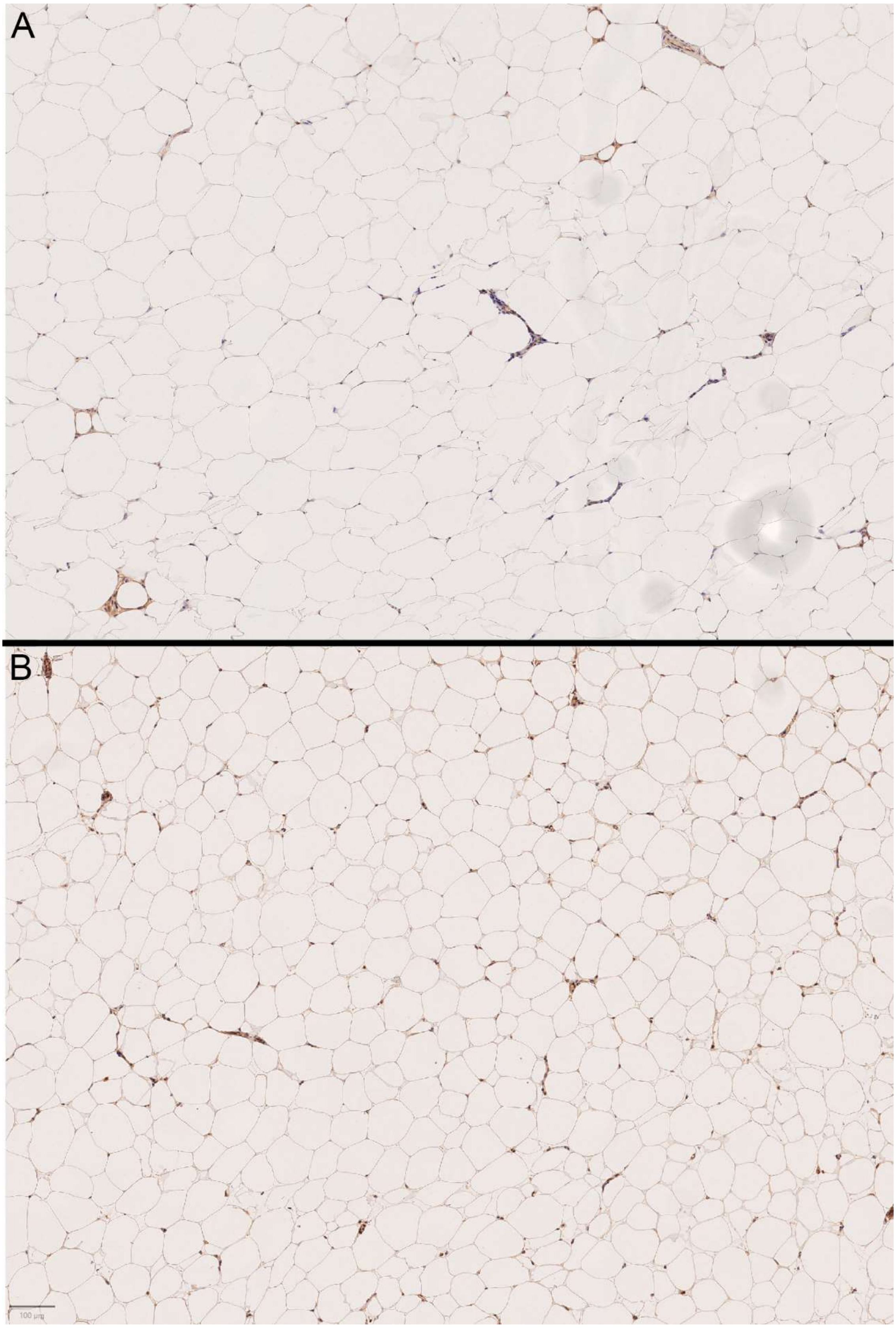

Supplementary Figure. Immunohistochemical staining of subcutaneous adipose tissue samples from the same subject was performed at baseline (A) and one year after (B) bariatric surgery using Caspase-1 antibody. A decline in crown-like structure numbers was observed; however, Caspase-1 was still present in individual macrophages and in the endothelium of vascular structures. Scale bar 100 µm.
